# The survival benefit of metastasectomy for metastatic non-clear cell renal cell carcinoma: a retrospective cohort study

**DOI:** 10.1007/s00345-024-04973-8

**Published:** 2024-04-25

**Authors:** Jindong Dai, Ben He, Yaowen Zhang, Haoran Zhang, Xu Hu, Lijing Xu, Yuchao Ni, Xingming Zhang, Guangxi Sun, Hao Zeng, Pengfei Shen, Zhenhua Liu

**Affiliations:** 1 Department of Urology, Institute of Urology, West China Hospital, Sichuan University, Chengdu, 610041 China; 2 Department of Urology, The Third People’s Hospital of Chengdu/The Affiliated Hospital of Southwest Jiaotong University, Chengdu, 610014 Sichuan China; 3 Department of Urology, Institute of Urology, West China Xiamen Hospital, Sichuan University, Xiamen, 361000 China

**Keywords:** Non-clear cell carcinoma, Surgery, Metastasectomy, Prognosis

## Abstract

**Purpose:**

The aim of this study was to explore the benefit the metastasectomy for patients with metastatic non-clear cell carcinoma (non-ccRCC).

**Methods:**

This study enrolled 120 patients with confirmed metastatic non-ccRCC from the RCC database of our center from 2008 to 2021. Patients without metastasectomy were grouped as radical nephrectomy without metastasectomy patients. The clinical outcomes included overall survival (OS) and progression-free survival (PFS). Cox regression and Kaplan–Meier analyses were used to assess potential factors that predict clinical benefits from metastasectomy.

**Results:**

A total of 100 patients received radical nephrectomy alone, while the remaining 20 patients underwent both radical nephrectomy and metastasectomy. There was no significant difference in age between the two groups. Out of 100 patients who underwent radical nephrectomy, 60 were male, and out of 20 patients who had both radical nephrectomy and metastasectomy, 12 were male. Patients who underwent systemic therapy plus radical nephrectomy and metastasectomy had significantly better PFS (27.1 vs. 14.0, *p* = 0.032) and OS (67.3 vs. 24.0, *p* = 0.043) than those who underwent systemic therapy plus radical nephrectomy alone. Furthermore, for patients without liver metastasis (*n* = 54), systemic therapy plus radical nephrectomy and metastasectomy improved both PFS (*p* = 0.028) and OS (*p* = 0.043). Similarly, for patients with metachronous metastasis, systemic therapy plus radical nephrectomy and metastasectomy improved both PFS (*p* = 0.043) and OS (*p* = 0.032). None of the patients experienced serious perioperative complications (Clavien–Dindo Classification ≥ III grade).

**Conclusion:**

Metastasectomy in patients with metastatic non-ccRCC may provide clinical benefits in terms of improved PFS and OS, especially in patients without liver metastasis and those with metachronous metastasis.

**Supplementary Information:**

The online version contains supplementary material available at 10.1007/s00345-024-04973-8.

## Introduction

Non-clear cell renal cell carcinoma (non-ccRCC) accounts for approximately 20–25% of all RCC cases and comprises close to 15 subtypes according to the 2022 World Health Organization classification [1]. Optimal management of metastatic non-ccRCC remains largely unknown [2]. The therapeutic strategies for metastatic non-ccRCC are largely extrapolated from clinical trials for ccRCC.

Although several clinical trials had explored the application of immune checkpoint inhibitors (ICIs) monotherapy or combined therapy for non-ccRCC [3], the cancer-specific survival in patients with metastatic non-ccRCC squint toward worse than metastatic ccRCC [4, 5]. KEYNOTE-564 trial has reported impressive results for the NED (M1 with no evidence of disease) subgroup, with a HR of 0.28 (0.12–0.66), indicating that metastasectomy may offer a survival benefit for certain patients with metastatic non-ccRCC. Several studies have suggested the important role of metastasectomy in patients with metastatic ccRCC [6–13]. Accordingly, the current European Association of Urology (EAU) guideline recommends metastasectomy for patients with metastatic disease and favorable clinical characteristics in whom total resection is possible [14]. Metastasectomy might be one of the options to achieve a complete and potentially durable cure in selected metastatic ccRCC patients with systemic therapies.

In the context of metastatic RCC studies, metastasectomy appears to be a viable option for metastatic non-ccRCC. The scarcity of data makes it difficult to determine the advantages of metastasectomy in patients with metastatic non-ccRCC. Could metastasectomy be a reliable therapy for patients with metastatic non-ccRCC? To our knowledge, no evidence supported the survival benefit of metastasectomy in those patients. The present study was conducted to evaluate the therapeutic efficacy of metastasectomy in patients with metastatic non-ccRCC.

## Methods

### Patient selection

Within the West China Hospital metastatic RCC database, we retrospective identified patients aged 18 years or older with metastatic RCC (International Classification of Disease for Oncology [ICD-O] site codes C64.9) who had been pathologically confirmed with non-clear cell histology between September 2008 and July 2021, and the included patients all had metastases with clear radiological or pathological diagnoses. Patients who were diagnosed before 2008 were excluded owing to incomplete information. And patients with metastatic non-ccRCC treated with systemic therapy plus nephrectomy. The systemic therapy regimens included tyrosine kinase inhibitors (TKIs) or mammalian target of rapamycin (mTOR) inhibitors, and the combination therapy regimens included TKIs combined with ICIs or mTOR. These selection criteria yielded 120 patients in our cohort. Out of the 120 patients in the study, 100 underwent nephrectomy only, whereas the remaining 20 patients received both nephrectomy and metastasectomy. Those who did not undergo metastasectomy were categorized as radical nephrectomy without metastasectomy patients. Furthermore, subgroup analysis was conducted based on the timing and location of metastasis. And this study has been registered in *researchregistry.com*; the Unique Identifying Number was researchregistry8996. The work has been reported in line with the STROCSS criteria [15].

### Features studied

Demographics included patient age at the first occurrence of distant metastasis, body mass index (BMI), and gender. Nephrectomy features included ECOG performance status, histological subtype, the International Metastatic RCC Database Consortium (IMDC) [16] risk score, clinical TNM stage [17], and surgical complications [18]. Pathology features included pathologic types of kidney tumor, the International Society of Urological Pathology (ISUP) [19]. Index metastasis features included timing to nephrectomy (synchronous, metachronous), number of distinct metastatic sites, location of metastases (pulmonary, bone, liver, or other), and whether the patient underwent metastasectomy.

For various treatment modalities, patients without metastasectomy were grouped as nephrectomy without metastasectomy patients.

### Follow-up and survival outcomes

Patients were suggested to accept outpatient follow-up at least once every three months and would increase the number of visits if needed. Telephone or email was used to contact patients who were unable to visit us to record their general condition and adverse events. Each routine follow-up included medical history records, physical examinations, and laboratory tests. Patients were required to conduct several imaging examinations (CT or MRI).

The clinical outcomes were progression-free survival (PFS) and overall survival (OS). PFS was defined as the time from metastasis to the first documented local or distant recurrence of renal cell carcinoma or death due to any cause. OS was defined as the time from metastasis to death due to any cause.

### Statistical analyses

PFS and OS rates were estimated using the Kaplan–Meier method. Associations with time to death from metastasectomy were assessed using Cox proportional hazards regression models and summarized with hazard ratios (HR) and 95% confidence intervals (CI). For multivariate analysis, clinical parameters were included in addition to those significant in univariate analyses. Statistical analyses were performed using SPSS, version 22. A *p* ≤ 0.05 was considered statistically significant.

## Results

### Patients’ characteristics

**Fig. 1:**
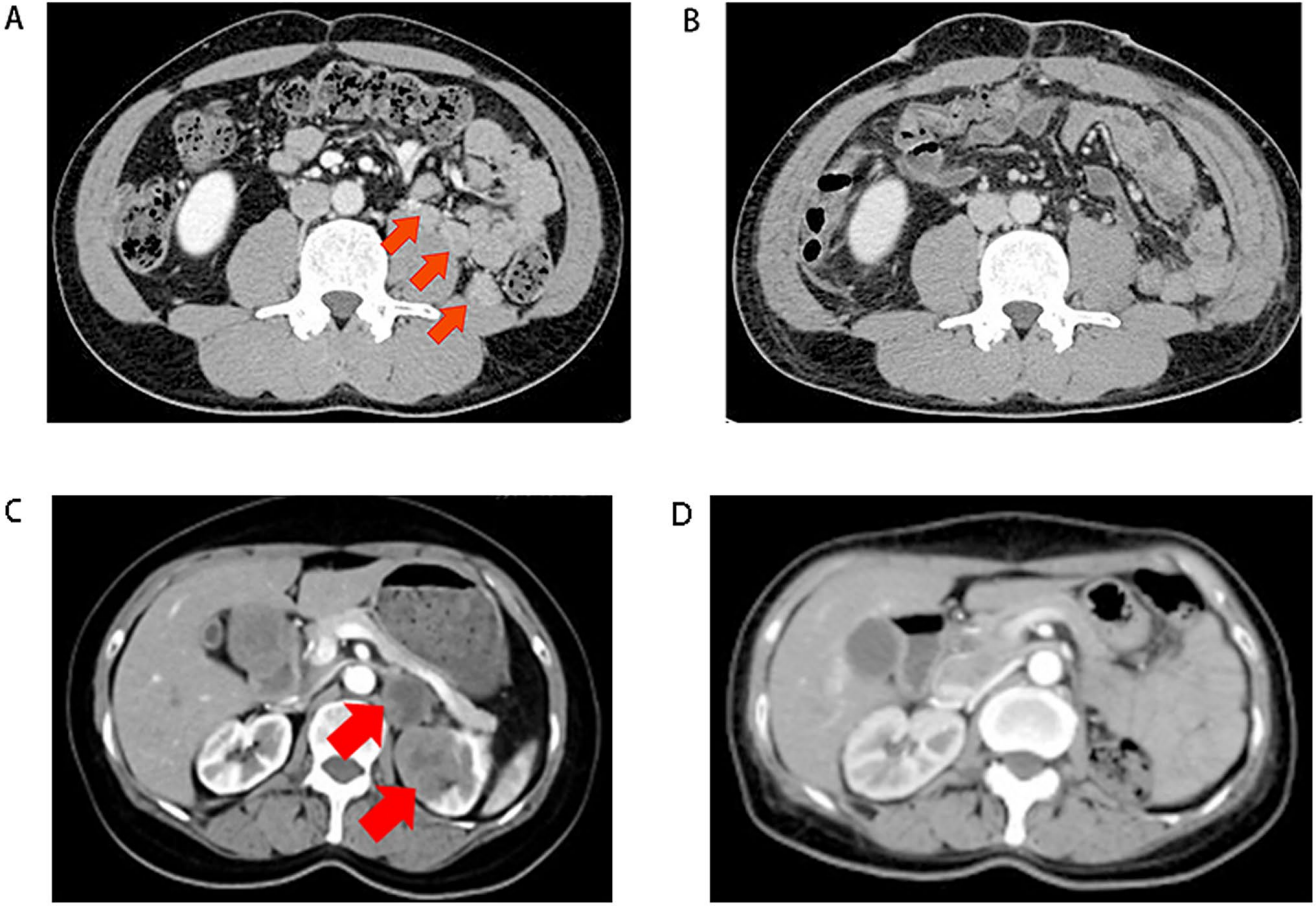
The CT scan of one of the patients underwent metastasectomy. **A** The preoperative CT scan of patients with metachronous metastasis who underwent metastasectomy. The arrow points to retroperitoneal metastasis lesions. **B** The postoperative CT scan of patients with metachronous metastasis who underwent metastasectomy. **C** The preoperative CT scan of patients with synchronous metastasis who underwent metastasectomy. The arrow points to retroperitoneal metastasis lesions. **D** The postoperative CT scan of patients with synchronous metastasis who underwent metastasectomy

We recruited 120 patients with metastatic non-ccRCC, of whom 20 (12.3%) received metastasectomy. The CT scans of some typical cases are shown in Fig. 1. The patients’ characteristics are shown in Table 1. Among 120 non-ccRCC patients, 19 (15.8%) patients had metastasis lesion in liver, and 4 of them underwent the metastasectomy; 30 (25.0%) patients had metastasis lesion in lung, and 3 of them received the metastasectomy. All the lymph node metastasis was distant. And a total of 35 metastatic lesions were dissected among 20 patients. Among the 120 patients, 61.7% (74/120) were pathologically diagnosed with papillary RCC (pRCC), 25.8% (32/120) were diagnosed with TFE3-RCC, and 9.2% (11/120) remained undetermined in terms of pathological classification. The clinical information of all patients received metastasectomy was listed in Supplementary Table 1.

**Table 1:** Baseline characteristic of metastatic non-ccRCC patients with different treatment strategies

	Nephrectomy with metastasectomy (NM) (*n* = 20)	Nephrectomy without metastasectomy (NM) (*n* = 100)	*P*
Age (Year, median)	36.0 (31.0–49.5)	45.0 (31.0–52.0)	–
*Gender*			0.875
Male	12 (60.0)	60 (60.0)	
Female	8 (40.0)	40 (40.0)	
*BMI*, *n* (%)			0.937
< 18.5	3 (15.0)	15 (15.0)	
18.5–25	7 (35.0)	31 (31.0)	
> 25	6 (30.0)	14 (14.0)	
Non-available	4 (20.0)	40 (40.0)	
*ECOG*, *n* (%)			0.176
< 2	18 (90.0)	77 (77.0)	
≥ 2	2 (10.0)	23 (23.0)	
*T stage*, *n* (%)			0.400
< 3	8 (40.0)	49 (49.0)	
≥ 3	9 (45.0)	42 (42.0)	
Unknown	3 (15.0)	9 (9.0)	
*IMDC grading*, *n* (%)			0.218
Low	1 (5.0)	11 (11.0)	
Intermediate	15 (75.0)	50 (50.0)	
High	3 (15.0)	12 (12.0)	
Unknown	1 (5.0)	27 (27.0)	
*Synchronous metastases*, *n* (%)			0.253
Yes	9 (45.0)	45 (45.0)	
No	11 (55.0)	55 (55.0)	
*Metastatic sites*, *n* (%)			–
Lung	3 (15.0)	27 (27.0)	
Bone	5 (25.0)	25 (25.0)	
Liver	4 (20.0)	15 (15.0)	
Brain	0	1 (1.0)	
Lymph node	15 (75.0)	36 (36.0)	
Others	13 (65.0)	36 (36.0)	
*No. metastatic sites*, *n* (%)			0.118
< 2	8 (40.0)	37 (37.0)	
≥ 2	12 (60.0)	63 (63.0)	
*No. metastatic lesions*, *n* (%)			0.041
≤ 2	9 (45.0)	24 (24.0)	
2–5	5 (25.0)	62 (62.0)	
> 5	6 (30.0)	14 (14.0)	
*Complete metastasectomy*			–
Yes	9 (45.0)	0	
No	11 (55.0)	100 (100.0)	
*No. excised metastases*			–
< 2	6 (30.0)	–	
≥ 2	14 (70.0)	–	
*Histopathology*, *n* (%)			0.844
pRCC	15 (75.0)	59 (59.0)	
ChRCC	0	0	
TFE3-RCC	3 (15.0)	29 (29.0)	
CDC	0	3 (3.0)	
Unclassified	2 (10.0)	9 (9.0)	
*ISUP*, *n* (%)			0.307
< 3	0	6 (6.0)	
≥ 3	16 (70.0)	70 (70.0)	
Unknown	4 (20.0)	24 (24.0)	
*1st therapy*, *n* (%)			–
TKIs	13 (65.0)	69 (69.0)	
TKIs + ICIs	7 (35.0)	26 (26.0)	
TKIs + mTOR	0	2 (2.0)	
mTOR	0	1 (1.0)	
ICIs	0	2 (2.0)	
Interval from diagnosis to metastasis, median (month)	15.2 (4.5–118.0)	12.2 (7.6–114.6)	–

*BMI* Body mass index, *ECOG* Eastern Cooperative Oncology Group, *IMDC* International mRCC Database Consortium, *ISUP* International Society of Urological Pathology, *RCC* Renal cell carcinoma, *pRCC* papillary *RCC*, *ChRCC* Chromophobe RCC, *CDC* Collecting duct carcinoma

All patients received primary systemic therapy, including 82 (68.3%) patients treated with TKI monotherapy and 33 (27.5%) with TKI + ICI combination therapy, 1 (0.8%) patient treated with mTOR monotherapy, 2 (1.7%) patients treated with TKI + mTOR combination therapy, 2 (1.7%) patients treated with ICI monotherapy (Supplementary Table 1). The median follow-up time from the diagnosis to metastasis was 12.5 months.

### Survival analysis in all patients

The findings from the univariate and multivariate Cox regression analyses for prognostic factors about PFS and OS are shown in Supplementary Table 2. In the univariate analysis, patients with metastasis lesions more than 2 (*p* = 0.010), and ECOG Score < 2 (*p* = 0.009) had longer PFS.

**Fig. 2:**
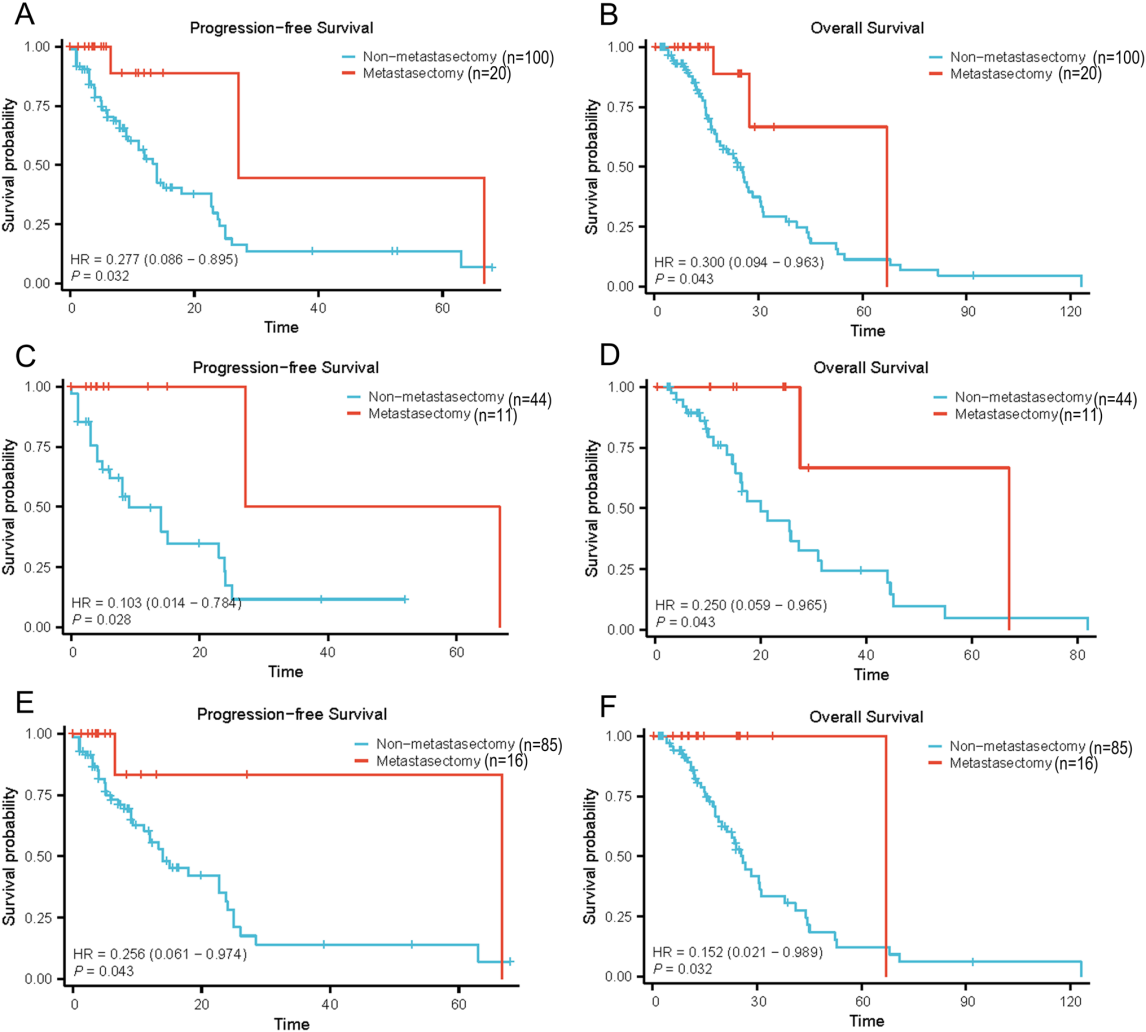
**A** The Kaplan–Meier curve estimating the PFS of patients who underwent metastasectomy. **B** The Kaplan–Meier curve estimating the OS of patients who underwent metastasectomy. **C** The Kaplan–Meier curve estimating the PFS of patients with metachronous metastasis based on undergoing metastasectomy. **D** The Kaplan–Meier curve estimating the OS of patients with metachronous metastasis based on undergoing metastasectomy. **E** The Kaplan–Meier curve estimating the PFS of patients without liver metastasis based on undergoing metastasectomy. **F** The Kaplan–Meier curve estimating the OS of patients without liver metastasis based on undergoing metastasectomy

In comparison between the metastasectomy(n = 20) and non-metastasectomy groups(*n* = 100), the PFS (27.1 vs. 14.0 months, HR: 0.277, 95%CI 0.086–0.895, *p* = 0.032) (Fig. 2A) and the OS (67.3 vs. 24.0 months, HR: 0.300, 95%CI 0.094–0.963, *p* = 0.043) (Fig. 2B) of patients who received metastasectomy was significantly longer than those without metastasectomy, and in multivariate Cox regression analyses, patients with systemic therapy plus nephrectomy and metastasectomy had longer PFS (HR:0.274, 95%CI0.084–0.899, *P* = 0.033).

**Fig. 3:**
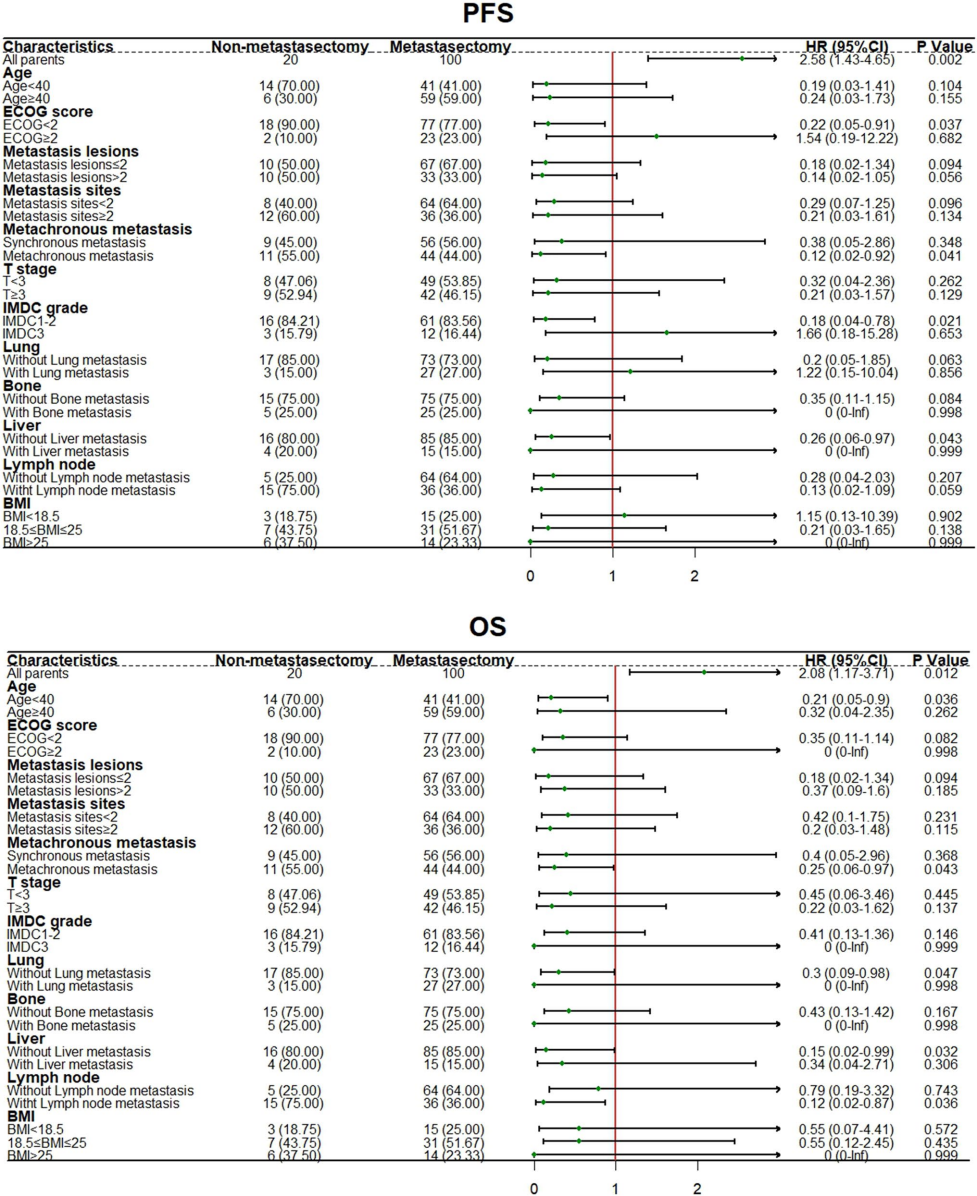
The forest plot of subgroup analysis. **A** The forest plot of subgroup analysis (PFS). **B** The forest plot of subgroup analysis (OS)

The appropriate candidate for metastasectomy was chosen based on subgroup analyses, with the corresponding results shown in Fig. 3.

### Survival analysis in the subgroup of patients with metachronous metastases

The duration from diagnosis to the occurrence of metastasis has played an important role in predicting patients’ prognosis [16]; therefore, we put much emphasis on the patients with metachronous metastases (*n* = 55) (Supplementary Table 3). The advantages of metastasectomy (*n* = 11) in PFS (HR: 0.103, 95%CI 0.014–0.784, *p* = 0.028) (Fig. 2C) and OS (HR: 0.250, 95%CI 0.059–0.965, *p* = 0.043) (Fig. 2D) were demonstrated in the univariate analyses, and in multivariate Cox regression analyses, patients with systemic therapy plus nephrectomy and metastasectomy had longer PFS (HR:0.117, 95%CI0.015–0.910, *p* = 0.040).

### Survival analysis in the subgroup of patients without liver metastasis

We further analyzed the importance of metastasectomy in patients without liver metastasis (*n* = 101) (Supplementary Table 4). Patients who received metastasectomy (*n* = 16) had significant longer PFS (HR: 0.256, 95%CI 0.061–0.974, *p* = 0.043) (Fig.2E) and OS (HR: 0.152, 95%CI 0.021–0.989, *P* = 0.032) (Fig. 2F).

### Perioperative complications

Supplementary Table 5 provides a detailed report of perioperative complications that each patient encountered. The median duration of the operation was 100.0 min (ranging from 73.0 to 132.5 min), and the median perioperative blood loss observed was 30.0 ml (ranging from 13.75 to 50 ml). Furthermore, patients spent a median hospital stay of 6.5 days (ranging from 6 to 8 days). Out of the total, 2 patients (10.0%) experienced postoperative fever, while 5 patients (25.0%) reported pain after the procedure. Additionally, only 1 patient (5.0%) suffered from a surgical site infection. It is important to note that none of the patients experienced any serious perioperative complications, based on the Clavien–Dindo Classification, with a grade ≥ III.

## Discussion

Non-clear cell renal cell carcinoma (non-ccRCC) is a diverse group of diseases with different histological traits, cellular origins, genomic properties, clinical features, clinical prognosis, and responses to therapy [20]. Renal tumors with papillary features represent a substantial proportion of cases sent for consultation. These include benign (e.g., papillary adenoma), indolent (e.g., clear cell papillary RCC), low malignant potential (e.g., eosinophilic solid and cystic RCC, mucinous tubular and spindle cell carcinoma, TCEB1-mutation RCC), malignant potential (e.g., papillary RCC, Tubulocystic RCC) and malignant and highly aggressive (e.g., collecting duct RCC, FH deficient RCC, TFE3-translocation RCC) [21]. Papillary RCC may also be a group of tumors with different driving genes, and FHRCC has been misdiagnosed as papillary RCC in many cases before, including the discovery by us and other centers that it may be driven by different genes such as NF2, STED2, and BAP1, and their different evolutionary trees may mean different benefits from tumor reduction. Previous randomized phase II trials have shown that Sunitinib (ESPN [22] and ASPEN [23]) provides better improvement in median PFS than Everolimus. However, more recent PAPMET [24] phase II trials have shown Cabozantinib is superior to Sunitinib in papillary RCC, but the median OS is merely 20 months, which is below the expected survival rate. Checkpoint inhibitor monotherapy (KEYNOTE-427) [25] or its combination with targeted therapy [26] has only marginally improved the median OS of metastatic non-ccRCC patients by a few months.

Next-generation sequencing (NGS) has been widely applied, allowed for a deeper understanding of variant genetic signatures and evolutionary trajectories of different subtypes of non-ccRCC. This highlights the need for more therapeutic strategies in addition to systemic treatments. KEYNOTE-564 trial has reported impressive results for the NED (M1 with no evidence of disease) subgroup, with a HR of 0.28 (0.12–0.66), indicating that metastasectomy may offer a survival benefit for certain patients with metastatic non-ccRCC. This study aimed to identify the benefits of metastasectomy for metastatic non-ccRCC, and the results suggest that both cytoreductive nephrectomy and metastasectomy can improve survival outcomes for patients with different metastatic non-ccRCC subtypes.

As with metastatic ccRCC, careful consideration must be given to whether metastasectomy is an appropriate intervention for patients with metastatic non-ccRCC. Since no randomized clinical trial has been conducted on this subject, it is difficult to identify the patient characteristics that might help predict the potential benefits of metastasectomy. Nonetheless, observational data suggest that favorable survival outcomes may be associated with a solitary disease site and complete resection using metastasectomy [27–30]. In addition, Suzuki et al. have found that patients with solitary metastasis of RCC who were treated with target therapy and metastasectomy have a significant advantage over that with target therapy alone in terms of OS, although the median OS of 62.9 months in the non-metastasectomy group was longer than the survival outcomes demonstrated in the phase III trial of target therapy for metastatic RCC. Regarding the results of the studies about metastatic RCC, metastasectomy in metastatic non-ccRCC seems feasible. However, in other studies, several patients were diagnosed with non-ccRCC in primary sites [27–30]. Due to the limited data available, it is challenging to identify the benefits of metastasectomy in patients with metastatic non-ccRCC.

For patients with metastatic disease, the timing of surgical intervention is also important. The clinical trial called SURTIME attempted to compare whether the timing of tumor reduction surgical interventions has a difference in clinical prognosis. The results of this trial showed that approximately 30% of patients who were scheduled for a reductive nephrectomy after preoperative drug therapy did not receive a reductive nephrectomy after systematic treatment. The most common reason for this is disease progression, suggesting that pre-tumor nephrectomy drug therapy may help select patients who are more likely to benefit from tumor reduction nephrectomy. These results may provide some reference for patients with metastatic non-ccRCC.

Moreover, following metastasectomy, 7 (50.0%) patients achieved NED. Of the patients who achieved NED, 85.7% (6/7) patients had non-visceral organ metastases, and all patients had an ECOG score of 0 before receiving metastasectomy (Supplementary Table 1). 57.1% (4/7) patients of patients who achieved NED had an IMDC score of less than 1. In contrast, for patients who did not receive metastasectomy, 38 (38.0%) patients had lesions in visceral organs, and 12 (12.0%) patients were identified of IMDC poor-risk subgroup. Based on the clinical characteristics of patients in our center, those with non-visceral organs and lower IMDC grade may be suitable for metastasectomy.

However, it is important to consider the technical intricacies and safety of surgery, in addition to therapeutic outcomes when implementing metastasectomy. A retrospective study indicated that among 1,102 patients who underwent metastasectomy, 27.5% experienced major complications (Clavien grade ≥ III), and liver metastasectomy being associated with a higher incidence of overall complications compared to procedures performed at other sites. In our cohort, all metastasectomic surgeries were safely performed through laparoscopic or robot-assisted approach. Four patients with relapse in situ, who had previous retroperitoneal surgical history, underwent reoperation with transperitoneal approach. Ten metastasectomies of relapse beside aorta or IVC did require more patience as well as exquisite skill to avoid major vessel injury. None of our patient had any serious perioperative complications, which suggested that the location of metastasis should be carefully considered when metastasectomy is planned, even in completely resectable cases.

The present study has several limitations that necessitate attention. Firstly, due to its retrospective design, this study comprised a relatively small patient population, thus making our data prone to selection bias. Secondly, despite adjusting our results for multiple relevant clinical and pathological factors, our findings may still be influenced by unexamined confounders and neglected values.

## Conclusion

In conclusion, metastasectomy may serve as a viable treatment option for patients with metastatic non-ccRCC. Individuals with metachronous metastases and those without liver metastases may benefit from metastasectomy. However, these findings require further exploration through extensive prospective cohort studies.

## Supplementary Information

Below is the link to the electronic supplementary material.

Supplementary file1 (DOCX 49 KB)

## Data Availability

The datasets used and analyzed during the current study are available from the corresponding author on reasonable request.
