# Supplementary material for: The survival benefit of metastasectomy for metastatic non-clear cell renal cell carcinoma: a retrospective cohort study
Source: World J Urol. 2024 Apr 25;42(1):259. doi: 10.1007/s00345-024-04973-8 (PMC11045608; doi:10.1007/s00345-024-04973-8)
Supplement: Supplementary file 1 — Supplementary file1 (DOCX 49 KB) [file 345_2024_4973_MOESM1_ESM.docx]

**Supplementary table 1. Clinical information of patients**

| Patients | Age  (Year) | Gender | Pathology  histology | Clinical  T stage | ECOG  score | Karnofsky  score | Neutrophils | Platelets | Ca>2.75 | Hb<120 | IMDC  Score | Site of metastasis | Resected lesion | Complete  resected | Systemic therapy | Tumor response |
| --- | --- | --- | --- | --- | --- | --- | --- | --- | --- | --- | --- | --- | --- | --- | --- | --- |
| 1 | 62 | Female | PRCC | 3a | 0 | 100 | 0 | 0 | 0 | 0 | 1 | Lymph node,  Retroperitoneal lesion | Lymph node,  Retroperitoneal lesion | Yes | SUN | NED |
| 2 | 68 | Male | Other | 3b | 3 | 70 | 0 | 0 | 0 | 1 | 3 | Lymph node, Lung | Lymph node | No | AXI | SD |
| 3 | 33 | Female | PRCC | 3a | 0 | 100 | 0 | 0 | 0 | 0 | 1 | Bone, Lung,  Lymph node, ovary  retroperitoneal lesion, | Lymph node, ovary,  retroperitoneal lesion, | No | AXI | SD |
| 4 | 31 | Male | PRCC | 1b | 0 | 100 | 0 | 1 | 0 | 0 | 2 | Bone, Lymph node | Lymph node | No | AXI +SIN | SD |
| 5 | 30 | Male | Other | 4 | 0 | 100 | 0 | 0 | 0 | 0 | 1 | Liver | Liver | No | EVE+SIN | SD |
| 6 | 26 | Male | PRCC | 2 | 2 | 100 | 0 | 1 | 0 | 1 | 3 | Bone,  Retroperitoneal lesion | retroperitoneal lesion | No | AXI +SIN | SD |
| 7 | 36 | Female | PRCC | 2a | 0 | 100 | 0 | 0 | 0 | 0 | 1 | Retroperitoneal lesion  Lymph node | Retroperitoneal lesion  Lymph node | Yes | AXI +SIN | NED |
| 8 | 31 | Male | TFE3-RCC | 2b | 0 | 100 | 0 | 0 | 0 | 0 | 0 | Lymph node | Lymph node | Yes | AXI | NED |
| 9 | 37 | Male | TFE3-RCC | 3b | 0 | 100 | 0 | 0 | 1 | 1 | 2 | Liver | Liver | No | AXI | SD |
| 10 | 27 | Female | TFE3-RCC | 3a | 0 | 100 | 0 | 0 | 0 | 0 | 1 | Retroperitoneal lesion  Lymph node | Retroperitoneal lesion  Lymph node | Yes | AXI +PEM | NED |
| 11 | 69 | Male | PRCC | - | 1 | 100 | 0 | 0 | 0 | 0 | 0 | Lymph node  Liver | Lymph node | No | AXI | SD |
| 12 | 37 | Female | PRCC | 2b | 0 | 100 | 0 | 1 | 1 | 0 | 2 | Paranephros  Lymph node | Paranephros  Lymph node | Yes | SUN | NED |
| 13 | 34 | Male | PRCC | 1b | 0 | 100 | 1 | 0 | 0 | 1 | 3 | Lymph node | Lymph node | Yes | AXI +SIN | NED |
| 14 | 27 | Male | PRCC | 1a | 0 | 100 | 1 | 0 | 0 | 1 | 2 | Retroperitoneal lesion | Retroperitoneal lesion | Yes | AXI | NED |
| 15 | 36 | Male | PRCC | 2b | 1 | 90 | 1 | 0 | 0 | 0 | 2 | Lung, Bone, Lymph node | Lymph node | No | AXI+SIN | SD |
| 16 | 51 | Female | PRCC | 3b | 1 | 90 | 0 | 0 | 0 | 0 | 1 | Retroperitoneal lesion | Retroperitoneal lesion | Yes | SUN | NED |
| 17 | 37 | Female | PRCC | 4 | 0 | 90 | 0 | 0 | 0 | 0 | 1 | Retroperitoneal lesion  Liver | Retroperitoneal lesion | Yes | SUN | NED |
| 18 | 45 | Female | PRCC | 2a | 1 | 90 | 0 | 0 |  | 0 | 1 | Retroperitoneal lesion | Retroperitoneal lesion | No | SUN | SD |
| 19 | 51 | Male | PRCC | 3b | 1 | 90 | 0 | 0 | 1 | 0 | 2 | Bone, Liver, Lymph node, Retroperitoneal lesion | Retroperitoneal lesion | No | SUN | SD |
| 20 | 35 | Male | PRCC | 2b | 1 | 90 | 0 | 0 | 1 | 0 | 2 | Retroperitoneal lesion, Lymph node, incision | Retroperitoneal lesion, incision | No | AXI+SIN | SD |

PRRCC: Papillary renal cell carcinoma; TKIs: tyrosine kinase inhibitors; ICIs: Immune checkpoint inhibitors; SD: stable disease; NED: no evidence of disease;

SUN: Sunitinib; AXI: Axitinib; PEM: Pembrolizumab; SIN: Sintilimab; EVE: Everolimus

**Supplementary table 2. Univariate analyses of factors associated with the PFS and OS among patients with metastasis non-ccRCC (n=120)**

|  | PFS | | | OS | | |
| --- | --- | --- | --- | --- | --- | --- |
|  | Univariate | | | Univariate | | |
|  | HR | 95%CI | P | HR | 95%CI | P |
| Age |  |  |  |  |  |  |
| <40 VS. ≥40 | 1.574 | 0.929-2.665 | 0.092 | 0.907 | 0.539-1.529 | 0.715 |
| Gender |  |  |  |  |  |  |
| Male vs. Female | 0.843 | 0.631-1.128 | 0.251 | 1.109 | 0.653-1.884 | 0.701 |
| BMI |  |  |  |  |  |  |
| Ref. | - | - | 0.142 | - | - | 0.524 |
| <18.5 VS. 18.5-25 | 0.687 | 0.254-1.859 | 0.460 | 0.719 | 0.325-1.592 | 0.416 |
| <18.5 VS. >25 | 0.398 | 0.158-0.999 | 0.050 | 0.599 | 0.243-1.480 | 0.267 |
| ECOG |  |  |  |  |  |  |
| <2 VS. ≥2 | 0.466 | 0.262-0.829 | 0.009 | 0.767 | 0.430-1.369 | 0.369 |
| T stage |  |  |  |  |  |  |
| <3 VS. ≥3 | 0.794 | 0.441-1.431 | 0.442 | 0.605 | 0.347-1.055 | 0.076 |
| IMDC grading |  |  |  |  |  |  |
| Ref. | - | - | 0.229 | - | - | 0.163 |
| Low VS. Intermediate | 0.202 | 0.023-1.754 | 0.147 | 0.207 | 0.024-1.773 | 0.412 |
| Low VS. High | 0.320 | 0.086-1.198 | 0.091 | 0.529 | 0.157-1.783 | 0.514 |
| Synchronous metastases |  |  |  |  |  |  |
| No VS. Yes | 1.232 | 0.712-2.130 | 0.455 | 1.405 | 0.834-2.368 | 0.202 |
| No. metastatic sites |  |  |  |  |  |  |
| <2 VS. ≥2 | 0.812 | 0.464-1.422 | 0.467 | 0.674 | 0.401-1.132 | 0.136 |
| No. metastatic lesions |  |  |  |  |  |  |
| ≤2 VS. >2 | 0.481 | 0.275-0.841 | 0.010 | 0.791 | 0.469-1.331 | 0.377 |
| ISUP |  |  |  |  |  |  |
| <3 VS. ≥3 | 0.260 | 0.061-1.111 | 0.069 | 0.479 | 0.147-1.560 | 0.222 |
| Metastasectomy |  |  |  |  |  |  |
| Yes vs. No | 0.277 | 0.086-0.895 | 0.032 | 0.300 | 0.094-0.963 | 0.043 |

BMI, Body mass index; ECOG, Eastern Cooperative Oncology Group; IMDC, International mRCC Database Consortium; ISUP, International Society of Urological Pathology;

**Supplementary table 3. Univariate analyses of factors associated with the PFS and OS among patients with metachronous metastases (n=55)**

|  | PFS | | | OS | | |
| --- | --- | --- | --- | --- | --- | --- |
|  | Univariate | | | Univariate | | |
|  | HR | 95%CI | P | HR | 95%CI | P |
| Age |  |  |  |  |  |  |
| <40 VS. ≥40 | 1.148 | 0.543-2.425 | 0.718 | 0.736 | 0.332-1.628 | 0.449 |
| Gender |  |  |  |  |  |  |
| Male vs. Female | 2.037 | 0.872-4.758 | 0.100 | 1.401 | 0.916-2.144 | 0.120 |
| BMI |  |  |  |  |  |  |
| Ref. | - | - | 0.790 | - | - | 0.175 |
| <18.5 VS. 18.5-25 | 1.119 | 0.244-5.130 | 0.884 | 4.228 | 0.898-19.901 | 0.068 |
| <18.5 VS. >25 | 0.683 | 0.161-2.894 | 0.605 | 2.714 | 0.713-10.335 | 0.143 |
| ECOG |  |  |  |  |  |  |
| <2 VS. ≥2 | 0.372 | 0.146-0.950 | 0.039 | 0.969 | 0.386-2.430 | 0.946 |
| T stage |  |  |  |  |  |  |
| <3 VS. ≥3 | 0.741 | 0.303-1.811 | 0.511 | 0.732 | 0.310-1.730 | 0.477 |
| IMDC grading |  |  |  |  |  |  |
| Ref. | - | - | 0.648 | - | - | 0.089 |
| Low VS. Intermediate | 0.461 | 0.091-2.331 | 0.953 | 0.081 | 0.007-1.041 | 0.054 |
| Low VS. High | 0.262 | 0.034-2.038 | 0.200 | 0.526 | 0.106-2.620 | 0.433 |
| No. metastasis |  |  |  |  |  |  |
| <2 VS. ≥2 | 0.704 | 0.306-1.621 | 0.409 | 0.706 | 0.326-1.527 | 0.377 |
| No. metastatic lesions |  |  |  |  |  |  |
| ≤2 VS. >2 | 0.461 | 0.198-1.072 | 0.072 | 0.515 | 0.234-1.131 | 0.098 |
| ISUP |  |  |  |  |  |  |
| <3 VS. ≥3 | 21.195 | 0.00-31.56 | 0.827 | 0.690 | 0.087-5.442 | 0.724 |
| Metastasectomy |  |  |  |  |  |  |
| Yes vs. No | 0.103 | 0.014-0.784 | 0.028 | 0.250 | 0.059-0.965 | 0.043 |

BMI, Body mass index; ECOG, Eastern Cooperative Oncology Group; IMDC, International mRCC Database Consortium; ISUP, International Society of Urological Pathology.

**Supplementary table 4. Univariate analyses of factors associated with the PFS and OS among patients** **without liver metastasis non-ccRCC (n=101)**

|  | PFS | | | OS | | |
| --- | --- | --- | --- | --- | --- | --- |
|  | Univariate | | | Univariate | | |
|  | HR | 95%CI | P | HR | 95%CI | P |
| Age |  |  |  |  |  |  |
| <40 VS. ≥40 | 1.674 | 0.891-3.147 | 0.110 | 1.123 | 0.601-2.099 | 0.716 |
| Gender |  |  |  |  |  |  |
| Male vs. Female | 0.570 | 0.289-1.124 | 0.105 | 0.972 | 0.709-1.333 | 0.861 |
| BMI |  |  |  |  |  |  |
| Ref. | - | - | 0.125 | - | - | 0.888 |
| <18.5 VS. 18.5-25 | 0.517 | 0.167-1.600 | 0.253 | 1.116 | 0.386-3.227 | 0.839 |
| <18.5 VS. >25 | 0.366 | 0.138-0.968 | 0.043 | 0.881 | 0.340-2.282 | 0.794 |
| ECOG |  |  |  |  |  |  |
| <2 VS. ≥2 | 0.516 | 0.260-1.022 | 0.058 | 0.633 | 0.313-1.279 | 0.202 |
| T stage |  |  |  |  |  |  |
| <3 VS. ≥3 | 0.807 | 0.417-1.564 | 0.525 | 0.455 | 0.232-1.891 | 0.062 |
| IMDC grading |  |  |  |  |  |  |
| Ref. | - | - | 0.612 | - | - | 0.390 |
| Low VS. Intermediate | 0.301 | 0.032-2.821 | 0.293 | 0.240 | 0.026-2.259 | 0.212 |
| Low VS. High | 0.569 | 0.134-2.419 | 0.445 | 0.642 | 0.151-2.721 | 0.547 |
| Synchronous metastases |  |  |  |  |  |  |
| No VS. Yes | 0.900 | 0.468-1.732 | 0.753 | 1.423 | 0.743-2.725 | 0.287 |
| No. metastatic sites |  |  |  |  |  |  |
| <2 VS. ≥2 | 0.817 | 0.418-1.594 | 0.553 | 0.671 | 0.354-1.274 | 0.223 |
| No. metastatic lesions |  |  |  |  |  |  |
| ≤2 VS. >2 | 0.591 | 0.304-1.146 | 0.120 | 0.978 | 0.517-1.850 | 0.946 |
| ISUP |  |  |  |  |  |  |
| <3 VS. ≥3 | 0.256 | 0.059-1.108 | 0.068 | 0.369 | 0.087-1.560 | 0.175 |
| Complete metastasectomy |  |  |  |  |  |  |
| Yes vs. No | 0.256 | 0.061-0.974 | 0.043 | 0.152 | 0.021-0.989 | 0.032 |

BMI, Body mass index; ECOG, Eastern Cooperative Oncology Group; IMDC, International mRCC Database Consortium; ISUP, International Society of Urological Pathology;

**Supplementary table 5. Perioperative complication in patients underwent metastasectomy**

|  | Clavien-Dindo Classification I-II  n, (%) | | | | | | Time of operation (Min) | Perioperative blood loss (ML) | length of hospital stays (Day) |
| --- | --- | --- | --- | --- | --- | --- | --- | --- | --- |
|  | Pain | Fever | Nausea | Oliguria | Constipation | Wound hematoma |  |  |  |
| Patient 1 | 1 | 0 | 0 | 0 | 0 | 0 | 160 | 70 | 7 |
| Patient 2 | 0 | 0 | 0 | 0 | 0 | 0 | 90 | 20 | 8 |
| Patient 3 | 0 | 0 | 0 | 0 | 0 | 0 | 105 | 50 | 6 |
| Patient 4 | 0 | 0 | 0 | 0 | 0 | 0 | 70 | 10 | 5 |
| Patient 5 | 0 | 0 | 0 | 0 | 0 | 0 | 150 | 50 | 6 |
| Patient 6 | 1 | 0 | 0 | 0 | 1 | 0 | 100 | 88 | 5 |
| Patient 7 | 0 | 0 | 0 | 0 | 0 | 0 | 120 | 30 | 10 |
| Patient 8 | 0 | 0 | 0 | 0 | 0 | 0 | 55 | 50 | 6 |
| Patient 9 | 0 | 0 | 0 | 0 | 0 | 0 | 130 | 60 | 9 |
| Patient 10 | 0 | 1 | 1 | 1 | 0 | 0 | 150 | 10 | 7 |
| Patient 11 | 0 | 0 | 0 | 0 | 0 | 0 | 120 | 20 | 6 |
| Patient 12 | 1 | 0 | 0 | 0 | 0 | 0 | 140 | 50 | 7 |
| Patient 13 | 1 | 0 | 0 | 0 | 0 | 1 | 200 | 30 | 8 |
| Patient 14 | 0 | 0 | 0 | 0 | 0 | 0 | 55 | 3 | 6 |
| Patient 15 | 0 | 0 | 0 | 0 | 0 | 0 | 95 | 25 | 6 |
| Patient 16 | 0 | 0 | 0 | 0 | 0 | 0 | 100 | 60 | 10 |
| Patient 17 | 0 | 1 | 0 | 0 | 0 | 1 | 70 | 10 | 7 |
| Patient 18 | 0 | 0 | 0 | 0 | 0 | 0 | 90 | 15 | 8 |
| Patient 19 | 0 | 0 | 0 | 1 | 0 | 0 | 74 | 30 | 6 |
| Patient 20 | 1 | 0 | 0 | 0 | 0 | 0 | 65 | 10 | 5 |
